# Supplementary material for: Glutamic acid is a carrier for hydrazine during the biosyntheses of fosfazinomycin and kinamycin
Source: Nat Commun. 2018 Sep 11;9:3687. doi: 10.1038/s41467-018-06083-7 (PMC6133997; doi:10.1038/s41467-018-06083-7)
Supplement: Supplementary file 1 — Supplementary Information [file 41467_2018_6083_MOESM1_ESM.pdf]

Supplementary Information

Glutamic acid is a carrier for hydrazine during the biosyntheses of fosfazinomycin and kinamycin

Wang *et al.*

Supplementary Figures

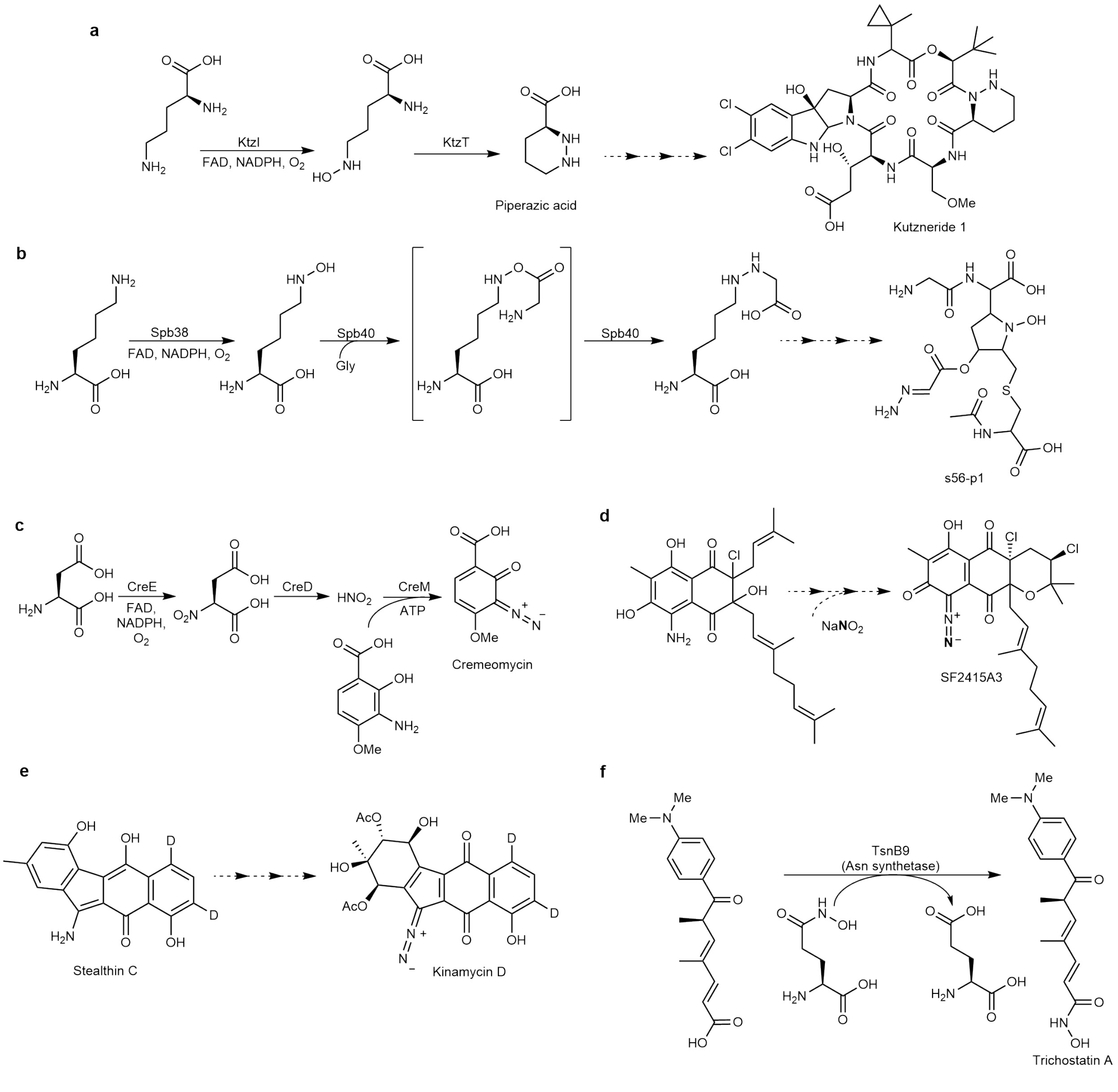

Supplementary Figure 1 Reconstituted or proposed biosynthetic pathways for the natural products discussed in the text. **(a)** A flavin-dependent enzyme (KtzI) activates ornithine for intramolecular attack catalyzed by KtzT to form the N–N bond during the biosynthesis of kutzneride[1, 2]. **(b)** In the biosynthesis of s56-p1, lysine is oxidized by Sbp38 and conjugated to glycine by Sbp40. Intramolecular attack from an amine then forms the N–N bond[3]. **(c)** During cremeomycin biosynthesis, nitrous acid is formed from aspartic acid before diazotization to form the N–N bond[4, 5]. **(d)** Labeling studies with ^{15}N-nitrite show incorporation of ^{15}N into the distal nitrogen in the diazo group of SF2415A3[6]. **(e)** Stealthin C was proposed to be an intermediate in kinamycin biosynthesis on the basis of a labeling study using deuterated stealthin C[7]. **(f)** TsnB9, an asparagine synthetase homolog, transfers hydroxylamine from the side chain of glutamic acid to complete the biosynthesis of trichostatin[8].

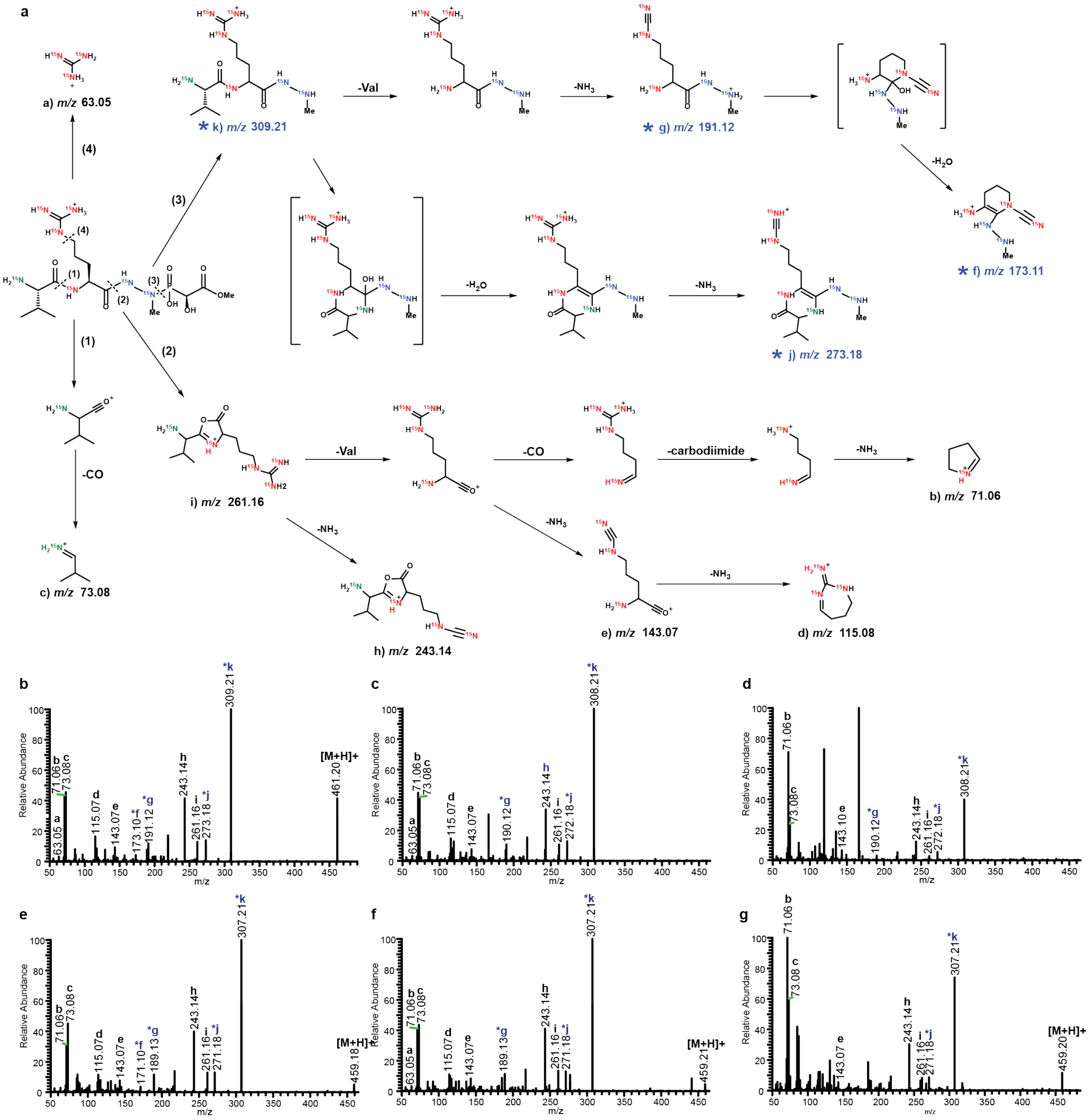

Supplementary Figure 2 MS/MS analysis of fosfazinomycin A from *Streptomyces* sp. NRRL S-149. **(a)** Proposed fragmentation pathway of uniformly ^{15}N-labeled fosfazinomycin with assigned ions. Product and fragment ions containing the N–N bond are indicated by asterisks and blue lettering. **(b)** MS/MS spectrum of uniformly ^{15}N-labeled fosfazinomycin A (precursor ion *m/z* 461.20). **(c, d)** MS/MS spectra of fosfazinomycin with one ^{14}N-incorporation (precursor ion *m/z* 460.20) from ^{15}N-labeled media with added **(c)** $NaNO_2$ or **(d)** *N*-hydroxyaspartic acid each at natural abundance (i.e. unlabeled). **(e, f, g)** MS/MS spectra of fosfazinomycin A with two ^{14}N-incorporations (precursor ion *m/z* 459.20) from ^{15}N-labeled media with added **(e)** hydrazinosuccinic acid, **(f)** acetylhydrazine, or **(g)** glutamylhydrazine (**6**), each at natural abundance. We note that in this experiment the primary amine on **6** is also unlabeled

(i.e. ^{14}N), and it is not possible to differentiate the fates of that nitrogen atom from the nitrogen atoms in the hydrazide functionality in **6**. However, we think that it is unlikely that the nitrogen atom of the primary amine of **6** is incorporated into both sites on the phosphonohydrazide linkage of fosfazinomycin given results from other feeding experiments and *in vitro* enzymatic assays.

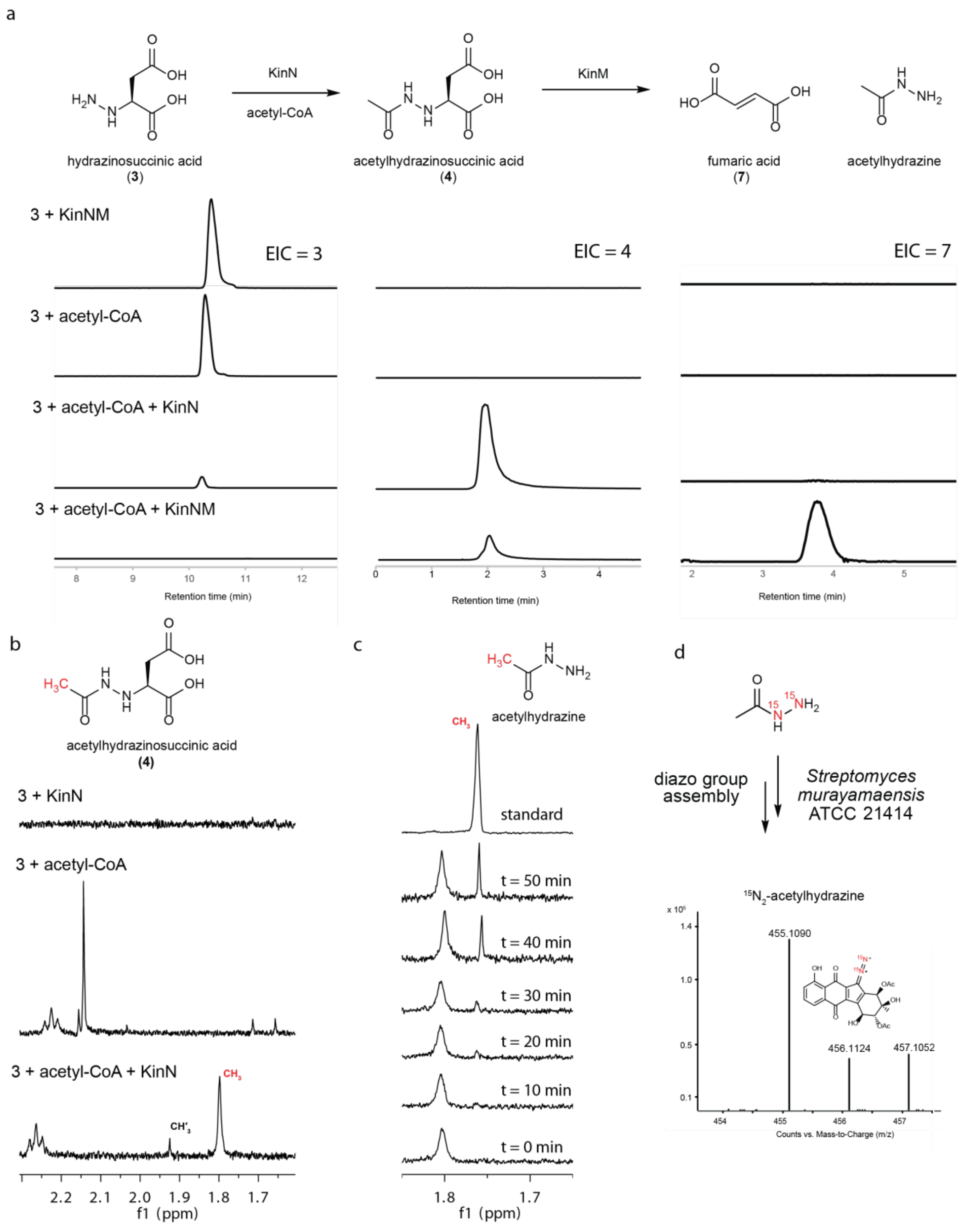

Supplementary Figure 3 *in vitro* generation of acetylhydrazine by KinNM and *in vivo* feeding experiment with acetylhydrazine. **(a)** EIC for hydrazinosuccinic acid (**3**, $[M-H]^-$ = 147.0411), *N*-acetylhydrazinosuccinic acid (**4**, $[M-H]^-$ = 189.0517), and fumaric acid (**7**, $[M-H]^-$ = 115.0037) of the reaction mixtures containing the components shown. **(b)** ^1H NMR spectrum of KinN assay mixtures containing the components shown. **(c)** ^1H NMR spectrum of acetylhydrazine generation from **4** with KinM. **(d)** HRMS of kinamycin D when *S. murayamaensis* is fed $^{15}N_2$-acetylhydrazine.

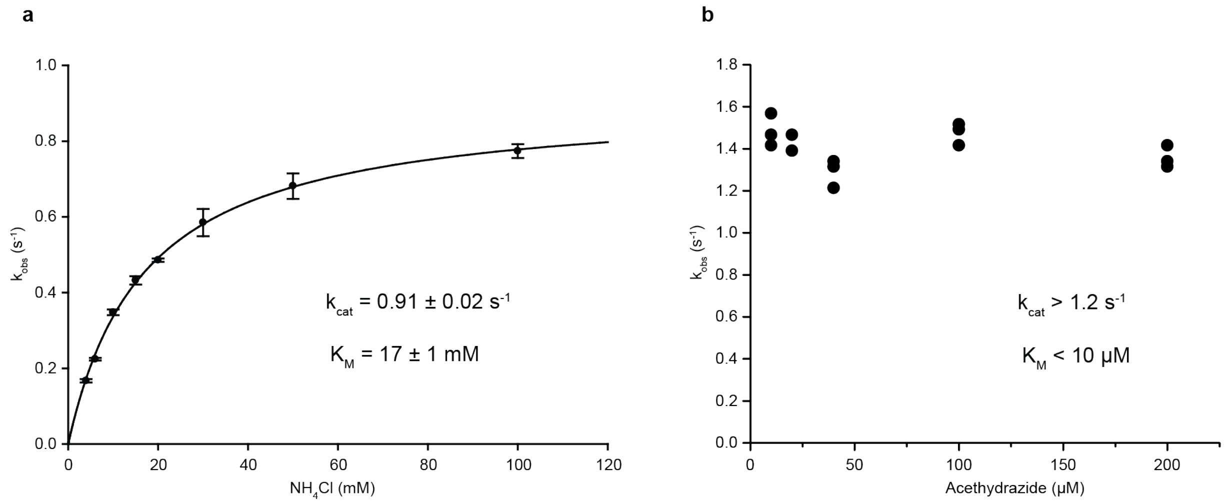

Supplementary Figure 4 Kinetic analysis of FzmN-catalyzed reactions. **(a)** Michaelis-Menten plot of FzmN-catalyzed Gln formation from Glu, NH_4Cl, ATP, and $MgCl_2$. The data points represent the average values obtained from triplicate experiments, and the error bars indicate the standard deviation. **(b)** Attempted kinetic analysis of the FzmN-catalyzed formation of glutamylacetylhydrazine from Glu, acetylhydrazine, NH_4Cl, ATP, and $MgCl_2$. Triplicate experiments are plotted.

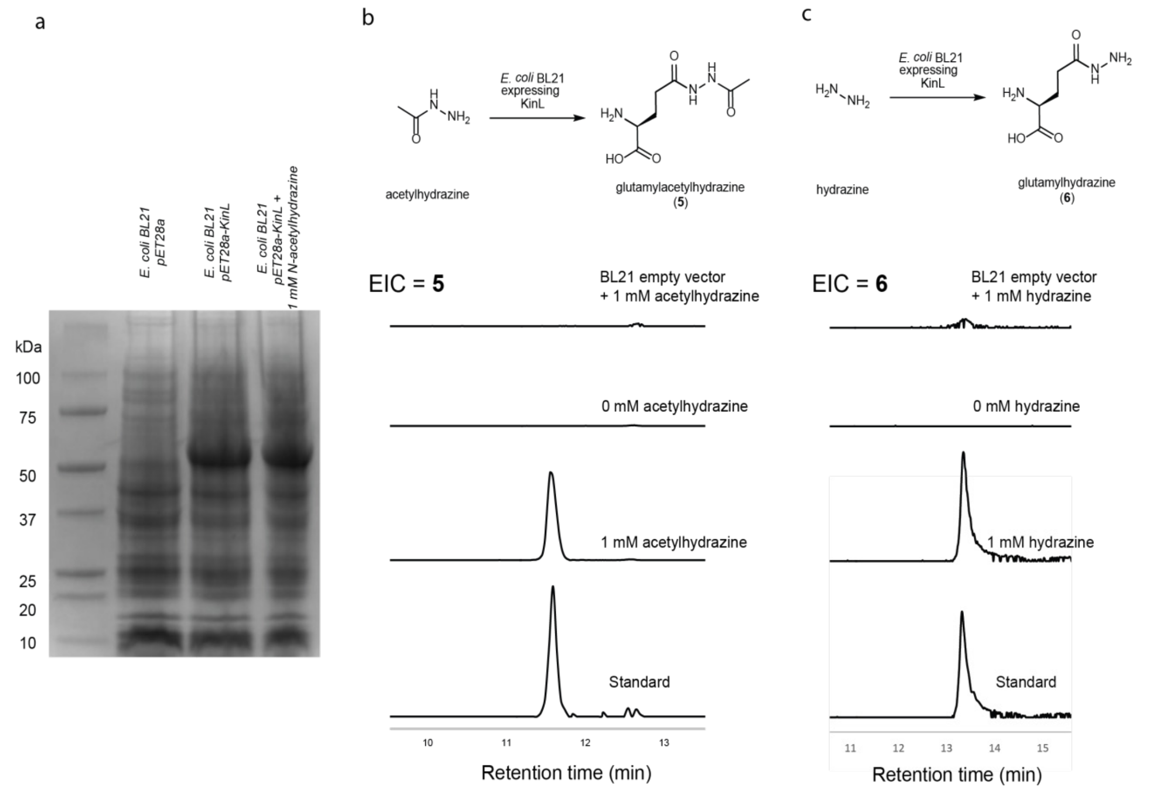

Supplementary Figure 5 *in vivo* activity assay of KinL. **(a)** SDS-PAGE of cell lysate demonstrating heterologous expression of KinL in *E. coli* BL21 (DE3) in the presence and absence of acetylhydrazine. The molecular weight for *N*-terminal His_6-tag KinL is 55.8 kDa. **(b)** KinL ligates acetylhydrazine to glutamic acid *in vivo* to afford **5**. EIC for **5** ($[M-H]^-$ = 202.0833). **(c)** KinL ligates hydrazine to glutamic acid *in vivo* to afford **6**. EIC for **6** ($[M-H]^-$ = 160.0728).

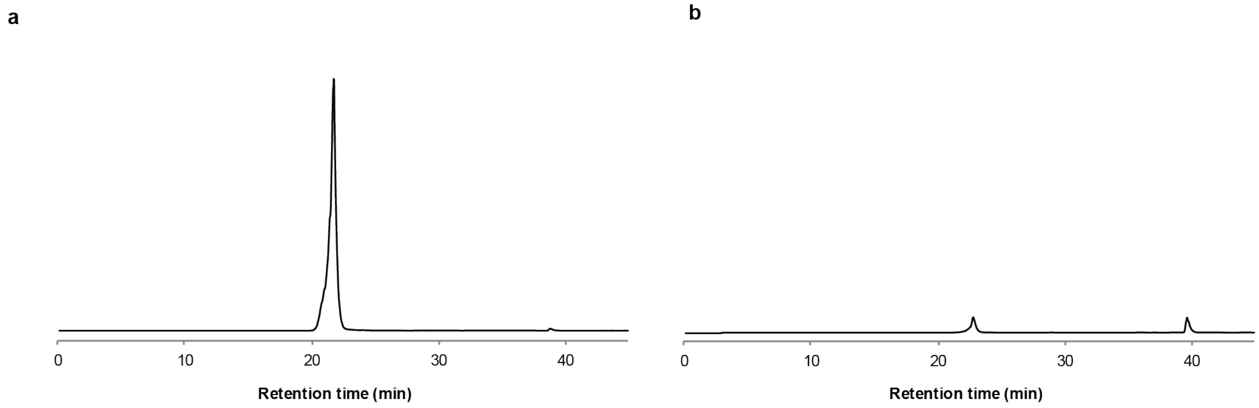

Supplementary Figure 6 LC-MS analysis of the FzmA-catalyzed reaction. The mass of Fmoc-derivatized glutamic acid ($[M-H]^-$ = 368) was monitored. **(a)** Chromatogram (selected ion monitoring for *m/z* 368) of the reaction after Fmoc-derivatization. **(b)** Chromatogram (selected ion monitoring for *m/z* 368) of the reaction mixture with FzmA omitted.

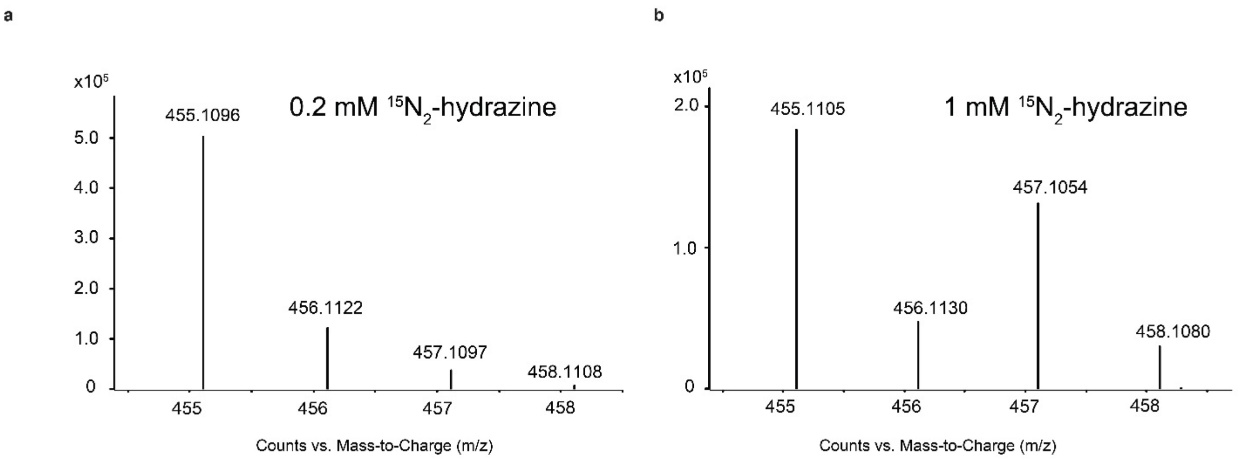

Supplementary Figure 7 HRMS analysis of $^{15}N_2$-hydrazine-labeled kinamycin D from *Streptomyces murayamaensis* ATCC 21414. HRMS analysis of ^{15}N incorporation into kinamycin D from feeding either **(a)** 0.2 mM or **(b)** 1 mM $^{15}N_2$-hydrazine to fermentation cultures of *S. murayamaensis*. $^{15}N_2$-kinamycin D ($[M+H]^+$ = 457.1026).

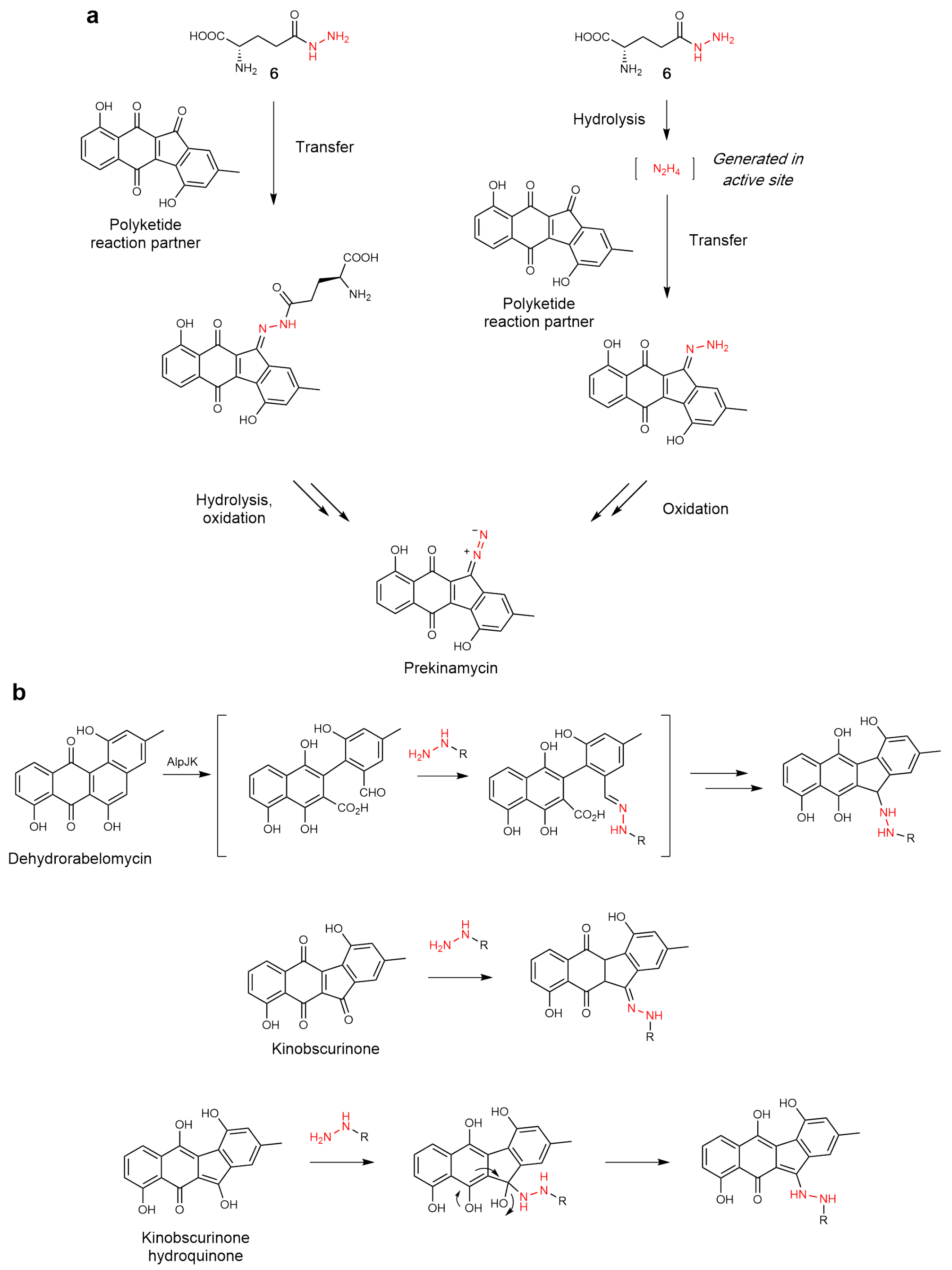

Supplementary Figure 8 Biosynthetic hypotheses for elaboration of **6** into the diazo group of the kinamycins. (**a**) Proposed logic for transfer of the hydrazine unit of **6** to a polyketide reaction partner. **(b)** Candidate polyketide reaction partners. R = H or glutamyl scaffold. See references for discussions of kinobscurinone[7] and dehydrorabelomycin[9].

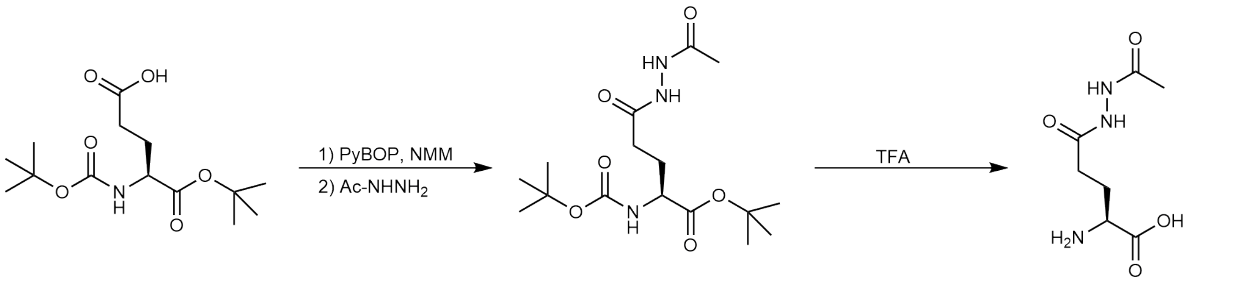

5a

Glutamylacetylhydrazine (**5**)

Supplementary Figure 9. The chemical synthesis of glutamylacetylhydrazine (**5**).

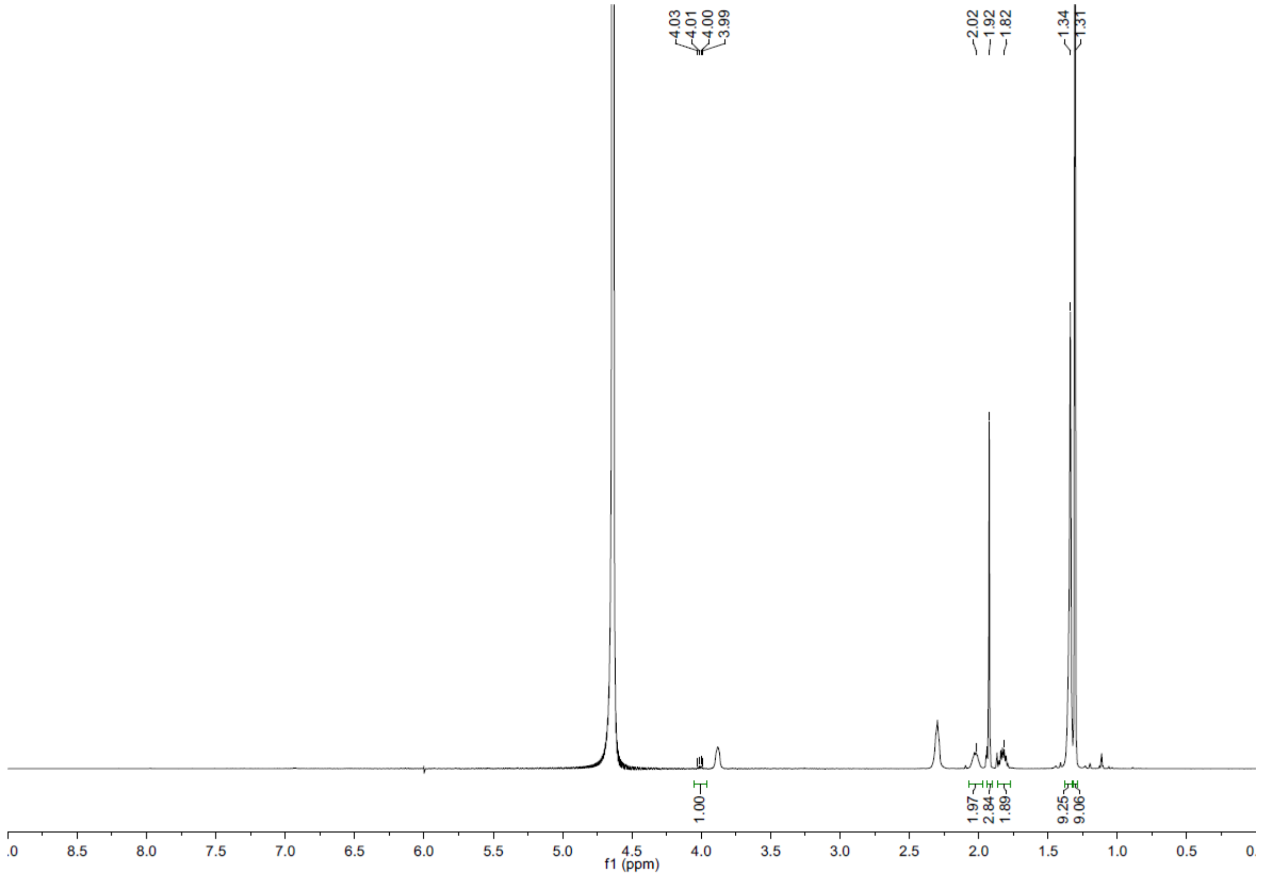

Supplementary Figure 10. ^{1}H NMR (600 MHz, D_2O) spectrum of **5a**.

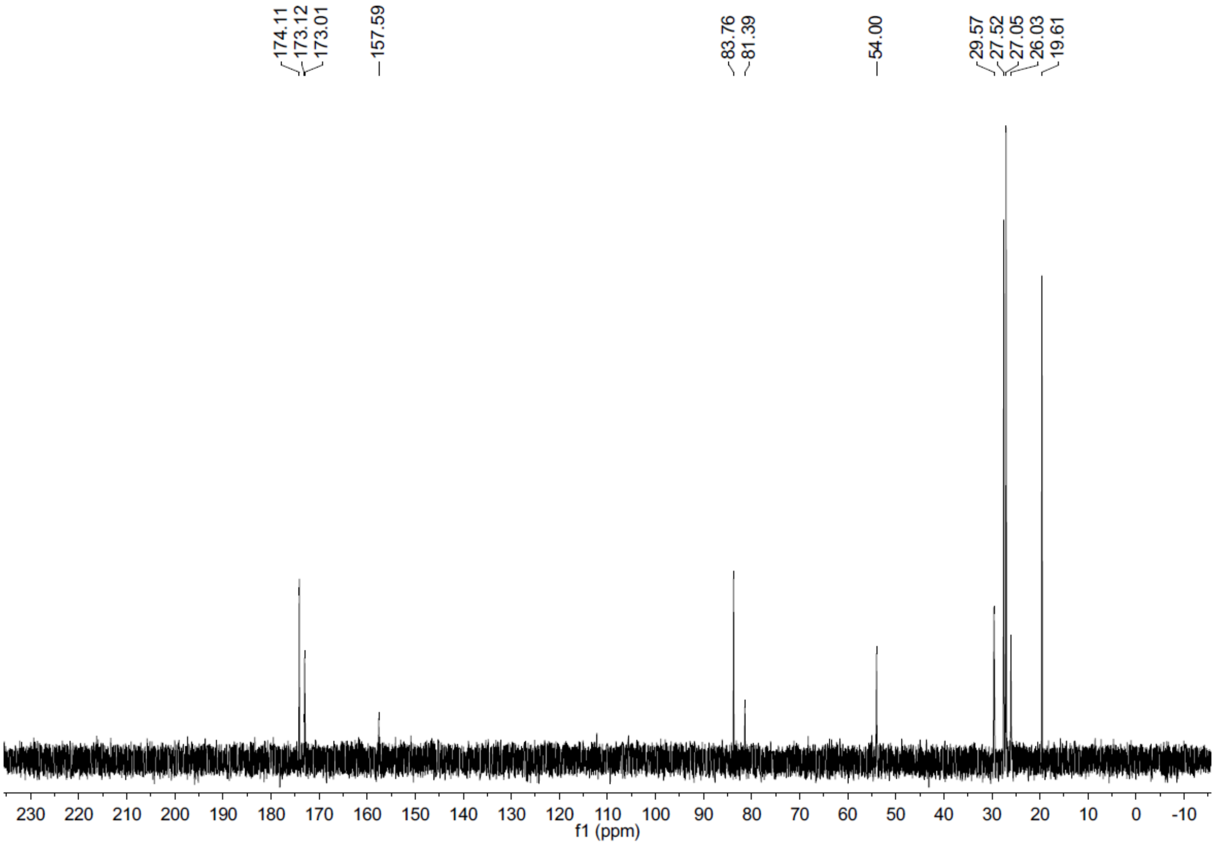

Supplementary Figure 11. ^{13}C NMR (150 MHz, D_2O) spectrum of **5a**.

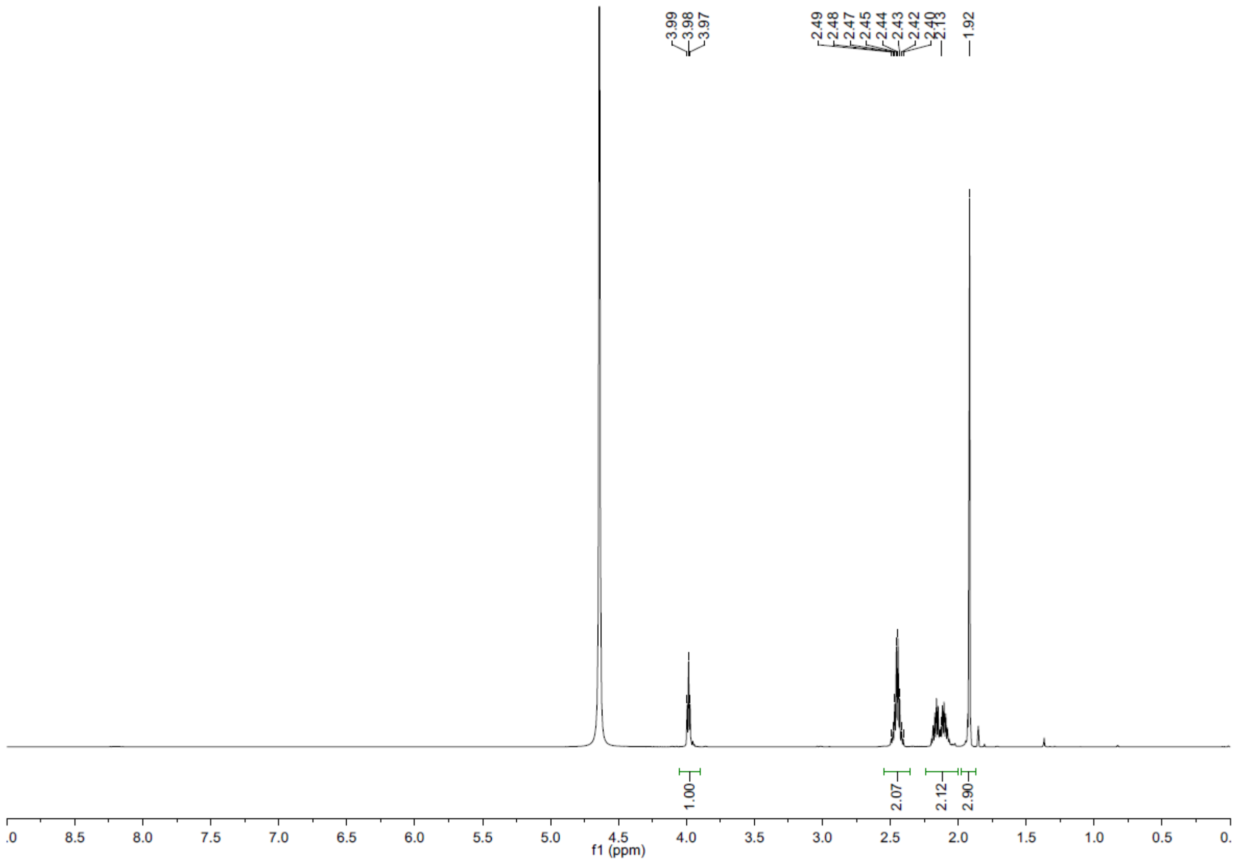

Supplementary Figure 12. ^{1}H NMR (600 MHz, D_2O) spectrum of **5**.

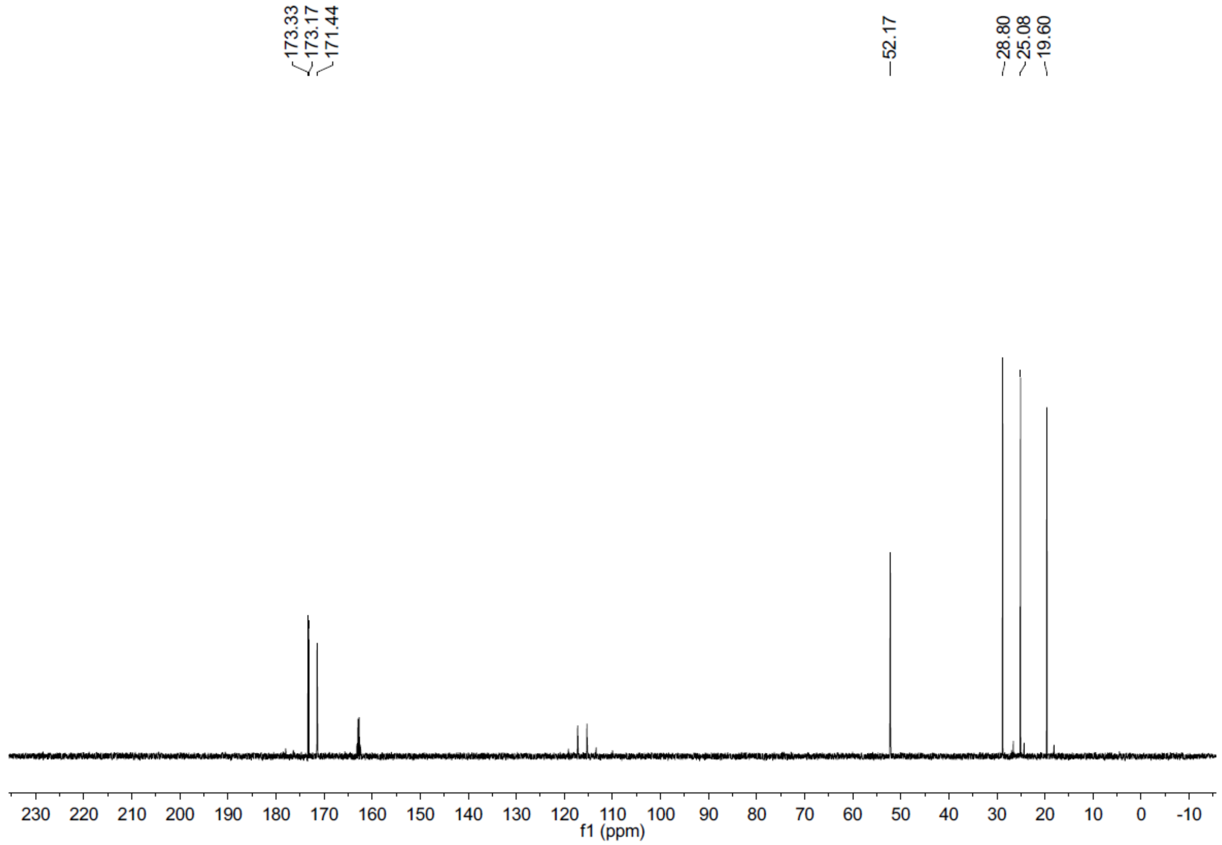

Supplementary Figure 13. ^{13}C NMR (150 MHz, D_2O) spectrum of **5**. The quartet at ~118 ppm and singlet at ~164 ppm are from residual TFA.

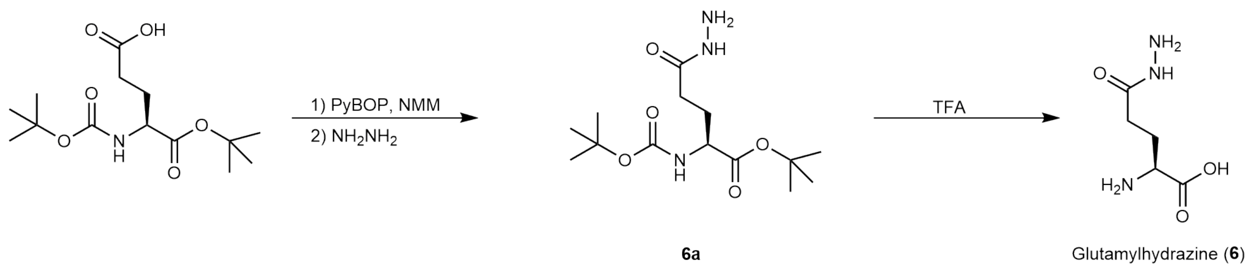

Supplementary Figure 14. The chemical synthesis of glutamylhydrazine (**6**).

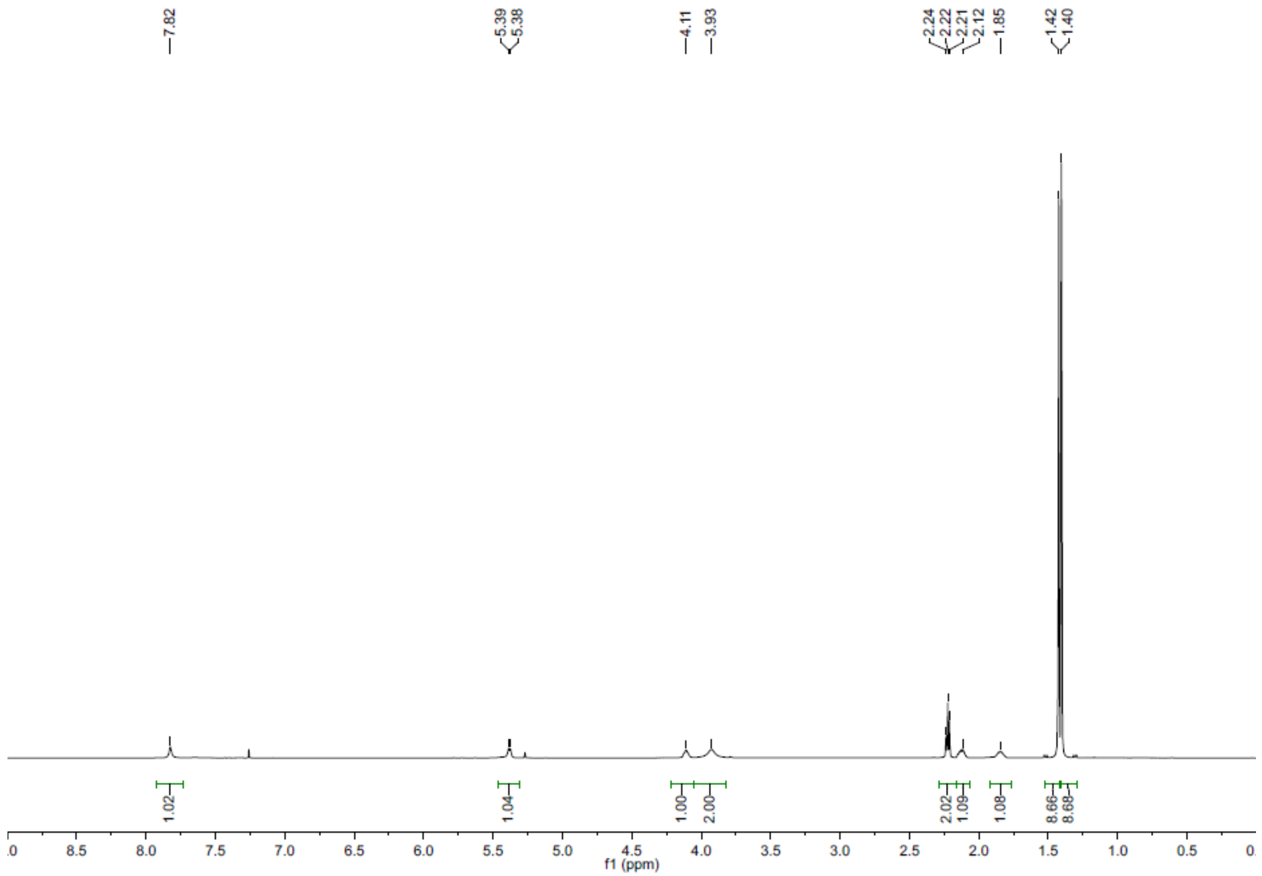

Supplementary Figure 15. ^{1}H NMR (600 MHz, $CDCl_3$) spectrum of **6a**.

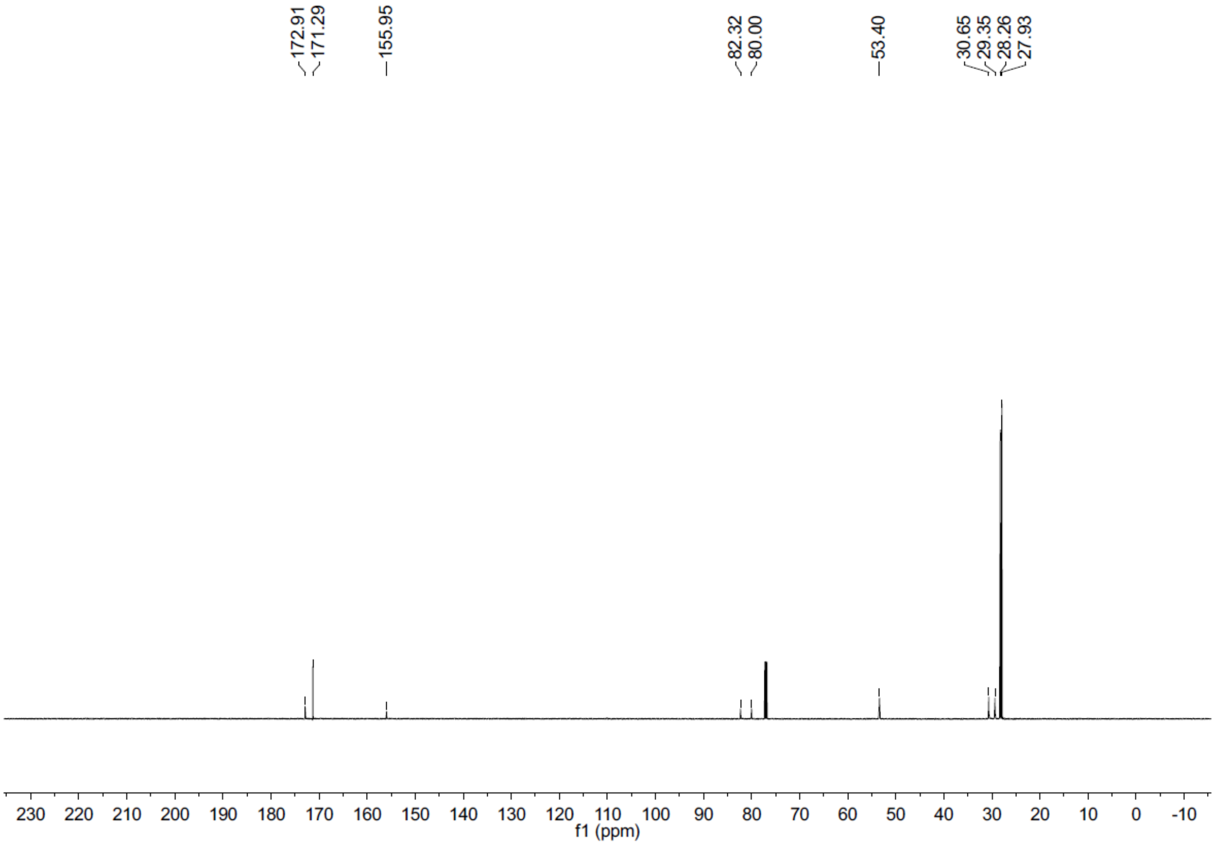

Supplementary Figure 16. ^{13}C NMR (150 MHz, $CDCl_3$) spectrum of **6a**.

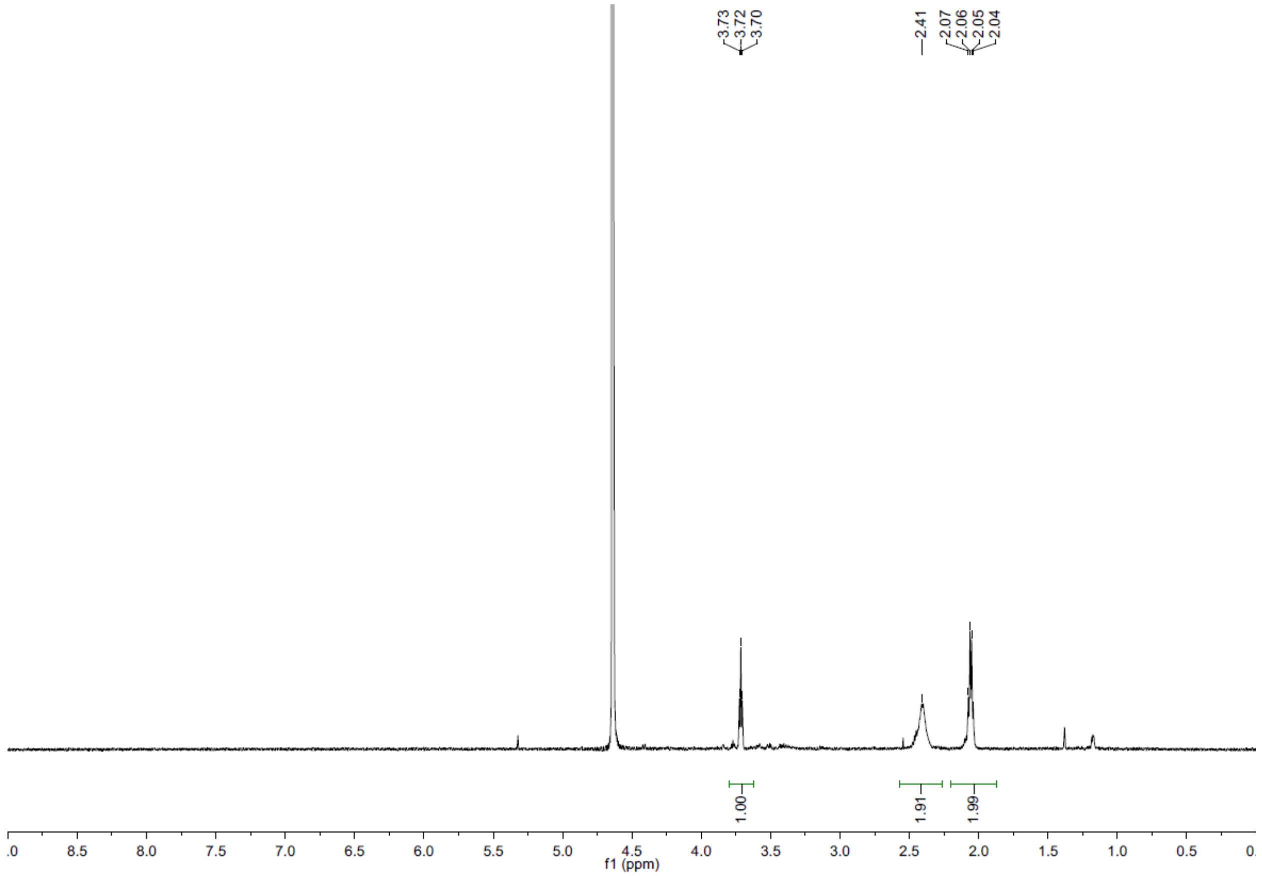

Supplementary Figure 17. ^{1}H NMR (600 MHz, D_2O) spectrum of **6**.

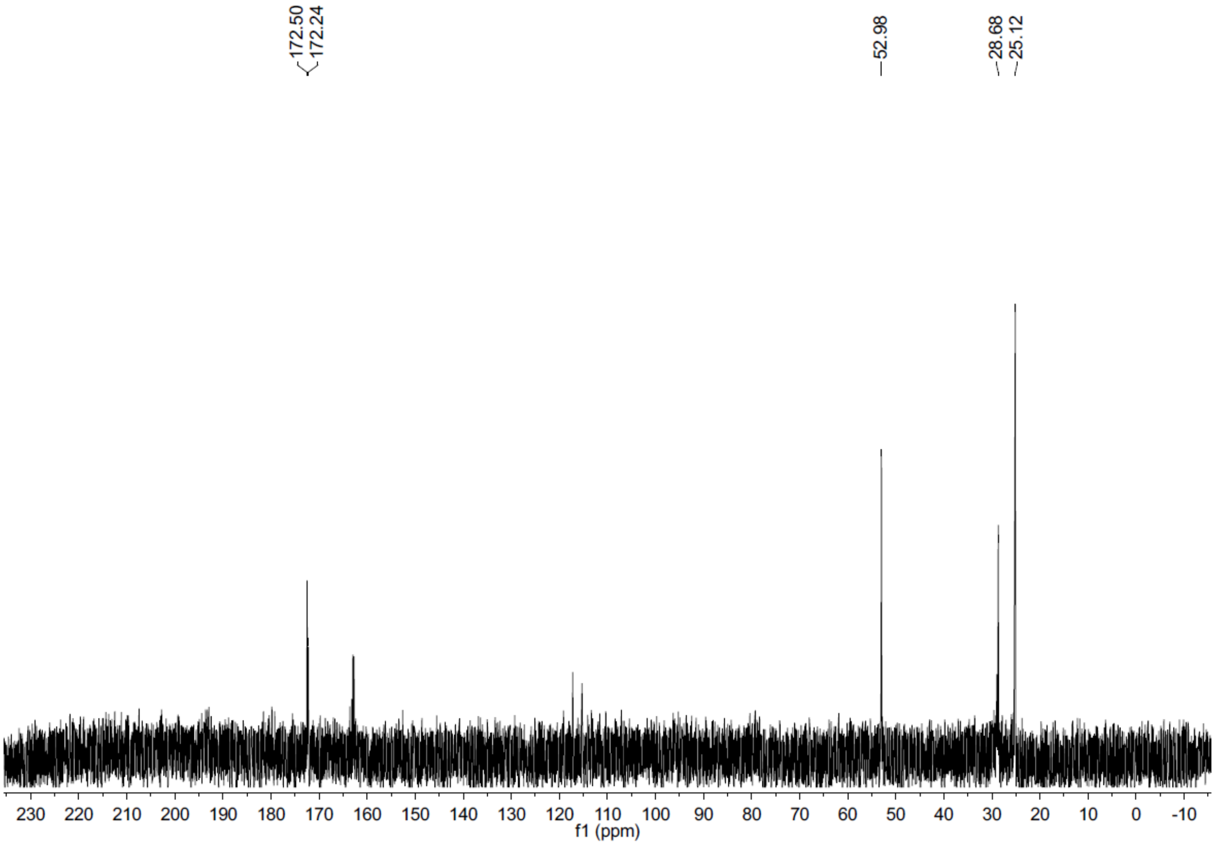

Supplementary Figure 18. ^{13}C NMR (150 MHz, D_2O) spectrum of **6**.

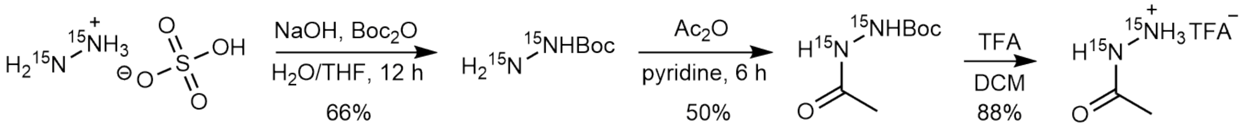

Supplementary Figure 19. The chemical synthesis of $^{15}N_2$-acetylhydrazine.

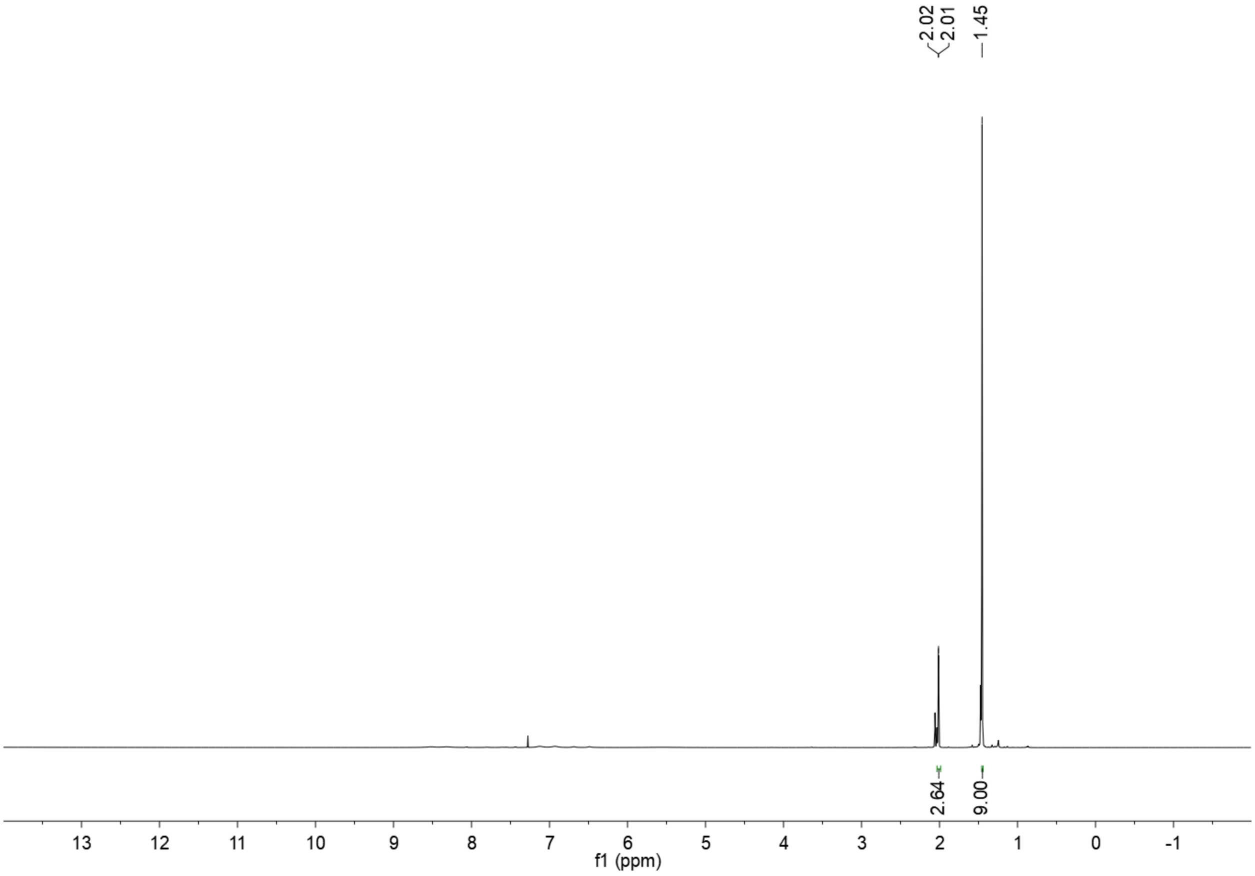

Supplementary Figure 20. ^{1}H NMR (500 MHz, $CDCl_3$) spectrum of $^{15}N_2$-Boc-acetylhydrazine.

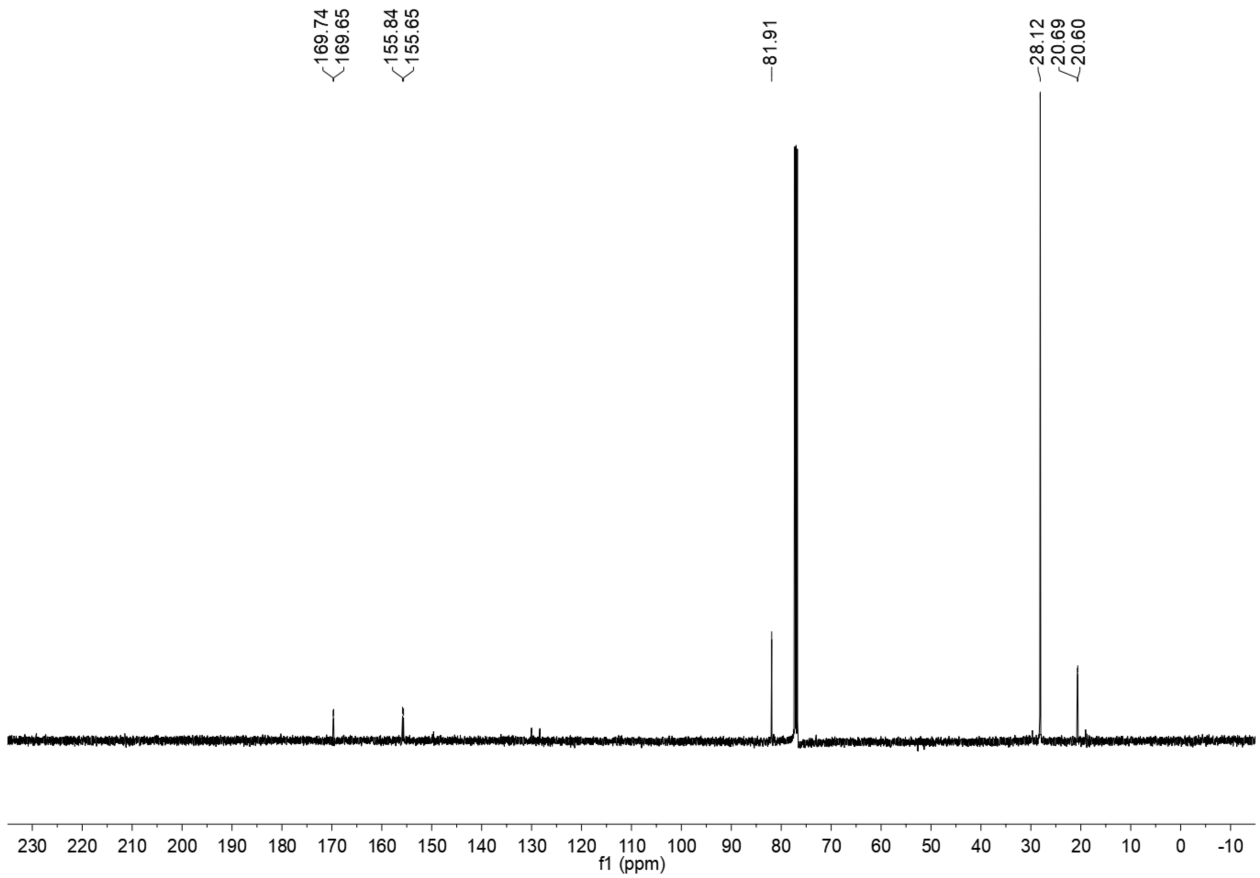

Supplementary Figure 21. ^{13}C NMR (125 MHz, $CDCl_3$) spectrum of $^{15}N_2$-Boc-acetylhydrazine.

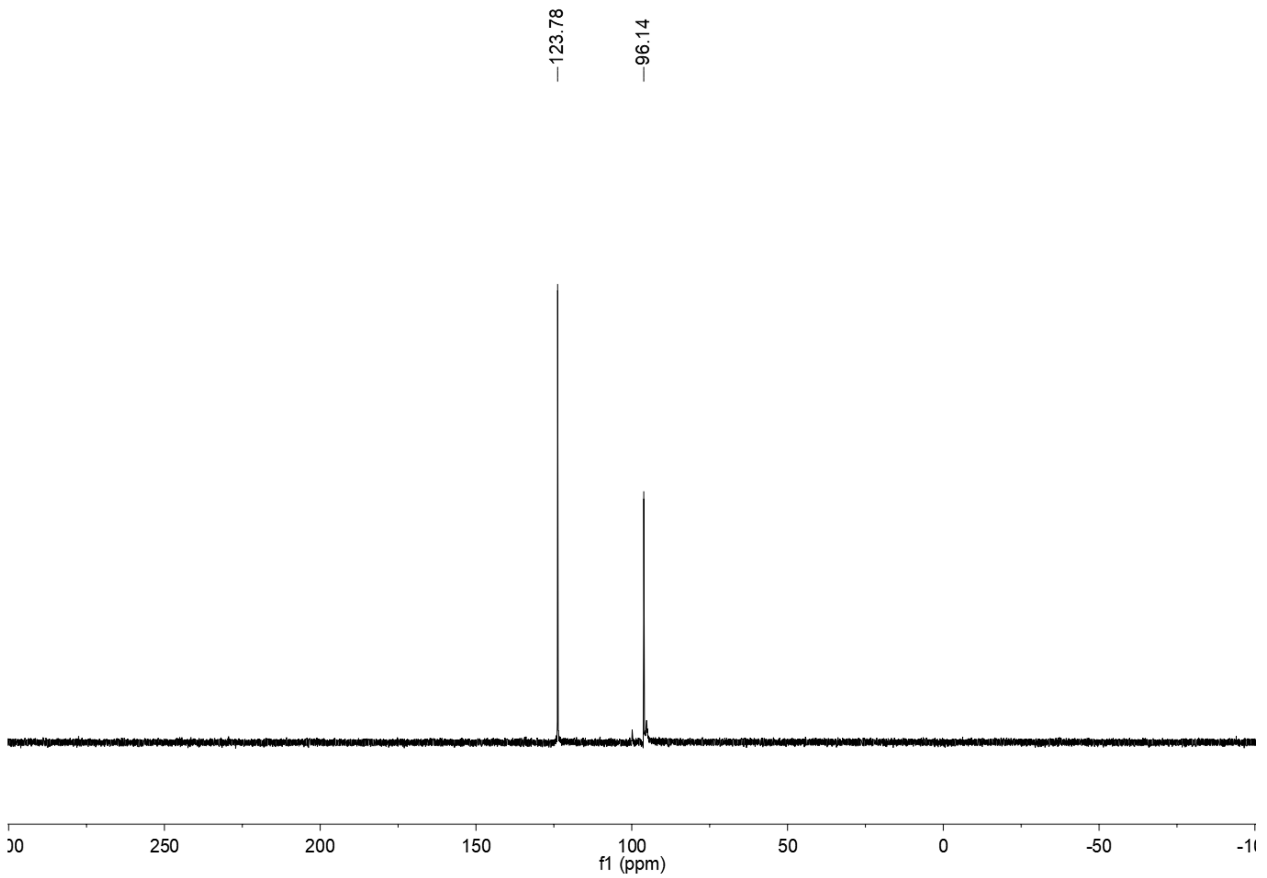

Supplementary Figure 22. ^{15}N NMR (41 MHz, $CDCl_3$) spectrum of $^{15}N_2$-Boc-acetylhydrazine.

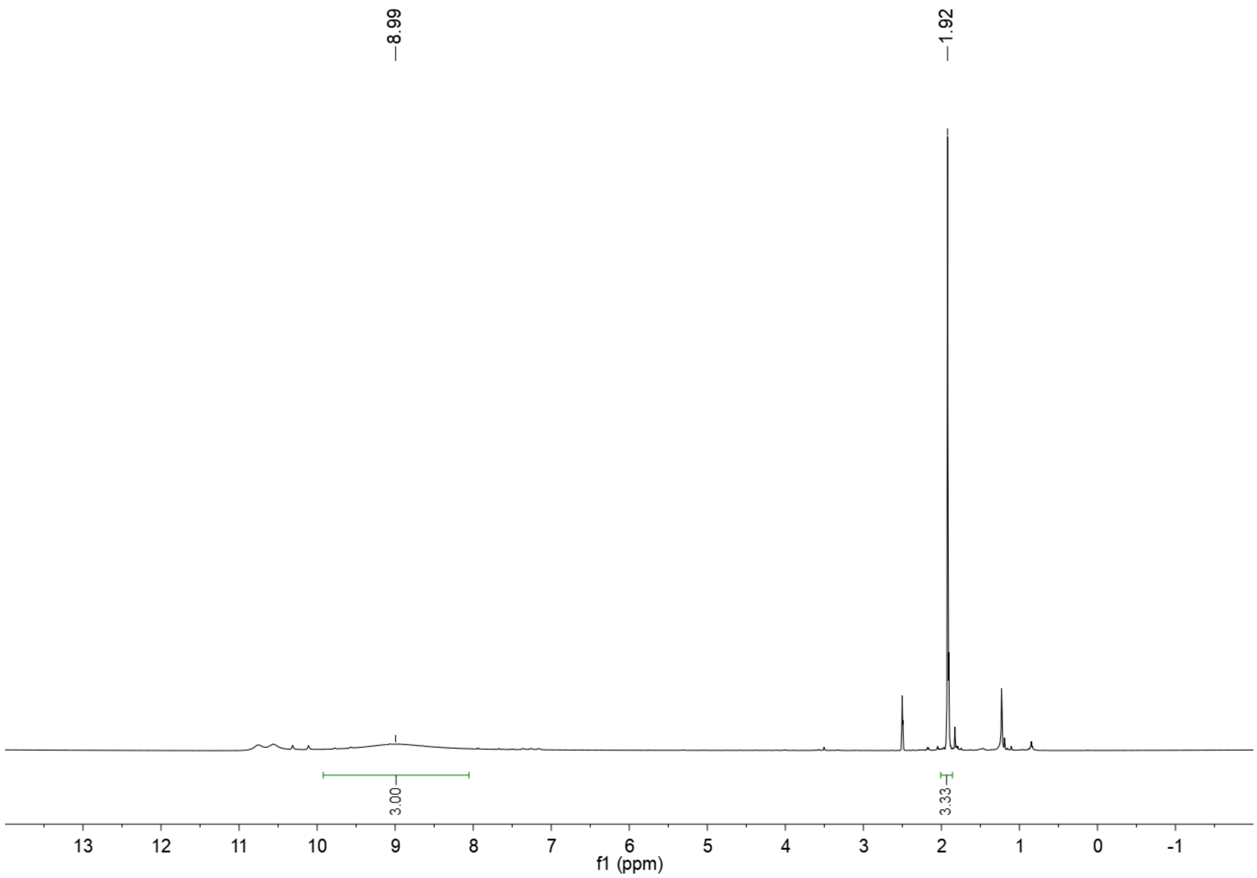

Supplementary Figure 23. ^{1}H NMR (500 MHz, d_6-DMSO) spectrum of $^{15}N_2$-acetylhydrazine.

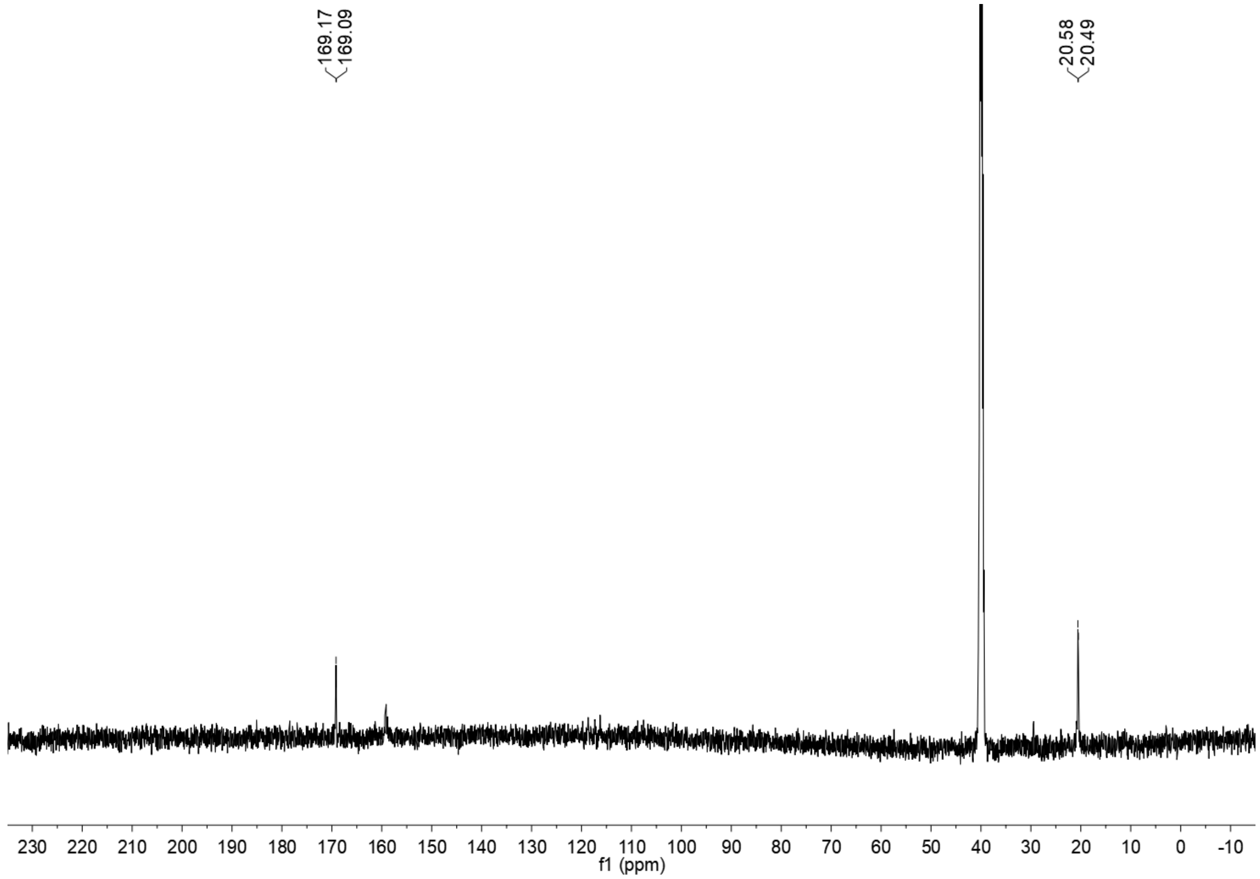

Supplementary Figure 24. ^{13}C NMR (125 MHz, d_6-DMSO) spectrum of $^{15}N_2$-acetylhydrazine.

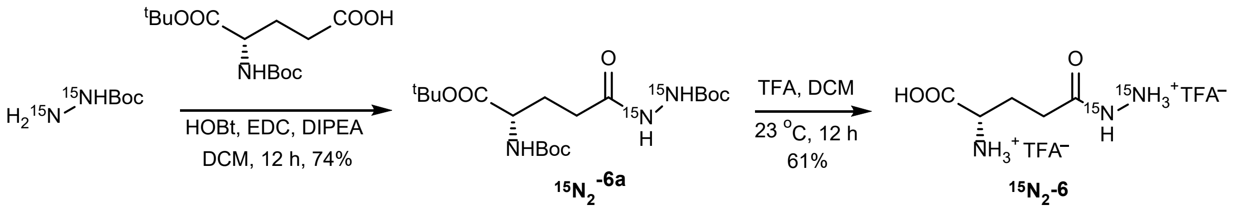

Supplementary Figure 25. The chemical synthesis of $^{15}N_2$-**6**.

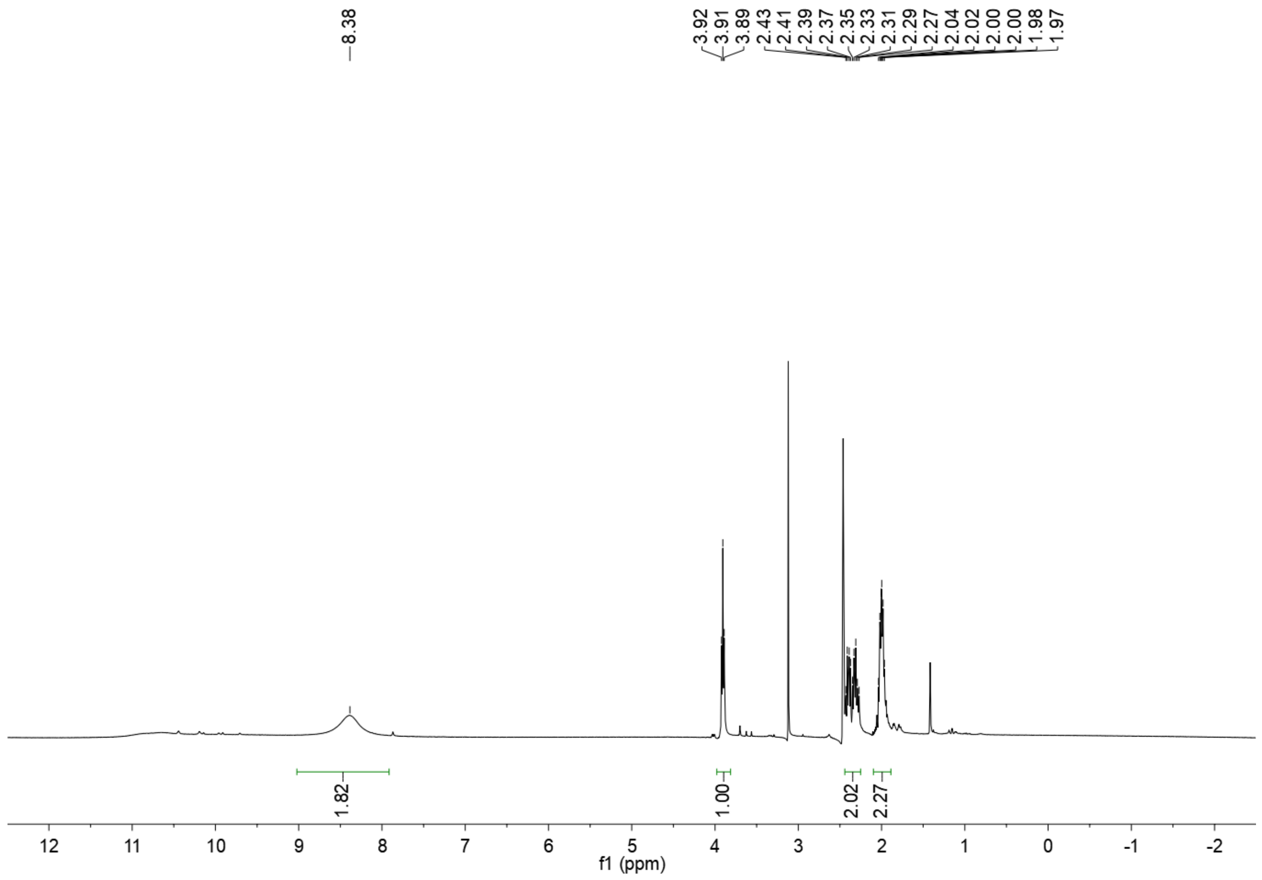

Supplementary Figure 26. ^{1}H NMR (400 MHz, d_6-DMSO) spectrum of $^{15}N_2$-**6**.

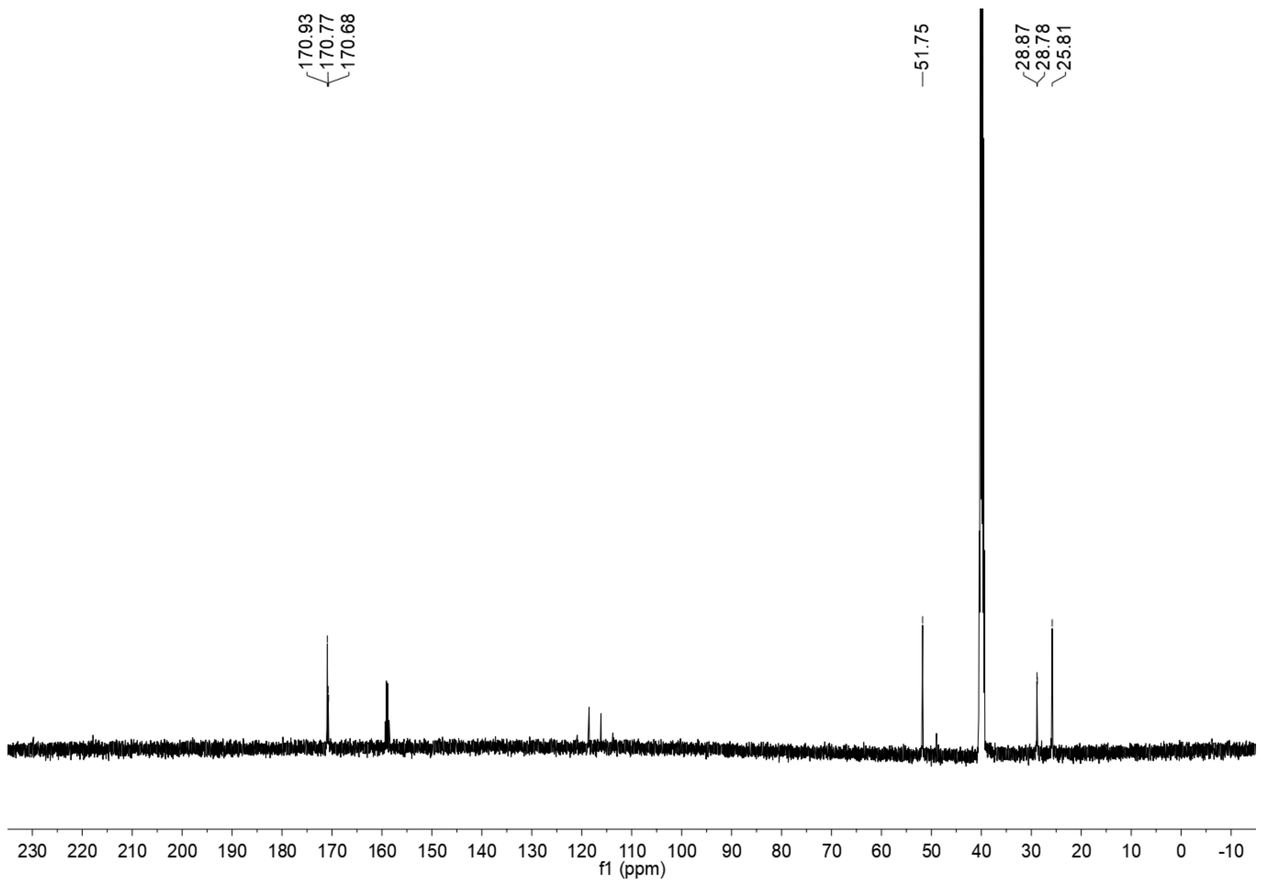

Supplementary Figure 27. ^{13}C NMR (125 MHz, d_6-DMSO) spectrum of $^{15}N_2$-**6**.

Supplementary Tables

Supplementary Table 1 Summary of the enzymes from fosfazinomycin biosynthesis (Fzm from *Streptomyces* sp. XY332) and kinamycin biosynthesis (Kin from *Streptomyces murayamaensis* ATCC 21414) discussed in the text. The sequence of each protein was queried by BLAST against the manually curated UniProtKB/Swiss-Prot database.

	**Uniprot/NCBI accession**	**Homologs from UniProtKB/Swiss-Prot database [%ID]**
FzmA	U5YQM4/ WP_053787804	Asparagine synthetase (*Bacillus subtilis* strain 168) [36.7%]
FzmL	U5YN81/ WP_053787793	3-carboxy-cis,cis-muconate cycloisomerase (*Pseudomonas aeruginosa* strain ATCC 15692) [42.7%]
FzmM	A0A0N0UQ79/ WP_053787792	NAD-dependent siroheme synthase (*Nitrosospira multiformis* strain ATCC 25196) [35%]
FzmN	A0A0M9CP55/ KOY56386	Glutamine synthetase (*Haloferax volcanii* strain ATCC 29605) [30.5%]
FzmO	U5YQN5/ WP_063785191	Putative amidase AF_1954 (*Archaeoglobus fulgidus* strain ATCC 49558) [38%]
FzmP	U5YN85/ WP_053787791	Transcription initiation factor TFIID subunit 4 (*Homo sapiens*) [45.1%]
FzmQ	U5YMA2/ WP_030299240	Mycothiol acetyltransferase (*Corynebacterium efficiens* strain DSM 44549) [38.2%]
FzmR	A0A0M9CPV0/ KOY56385	Adenylosuccinate lyase (*Bacillus subtilis*) [46.0%%]
KinJ	/ MH720502	DNA directed RNA polymerase subunit beta (*Nitrosospira multiformis* strain ATCC 25196) [41.1%]
KinK	/ MH720503	Putative amidase AF_1954 (*Archaeoglobus fulgidus* strain ATCC 49558) [33.9%]
KinL	/ MH720504	Glutamine synthetase (*Thermococcus kodakarensis* strain ATCC BAA-918) [30.5%]
KinM	/ MH720505	Adenylosuccinate lyase (*Synechocystic* sp.strain PCC 6803) [46.4%]
KinN	/ MH720506	Mycothiol acetyltransferase (*Corynebacterium efficiens* strain DSM 44549) [39.0%]
KinW	/ MH720500	Putative glutamine amidotransferase (*Streptomyces sp. TLI_146*) [93.2%]

Supplementary Table 2 Oligonucleotides and synthetic gene used for cloning. The primers are named for the amplicon and direction of amplification: forward (fwd or F) or reverse (rev or R); uppercase letters indicate portions that anneal to the amplicon while lowercase letters indicate the overhang regions for Gibson assembly

**Cloning primer**	**Sequence**
FzmN_fwd	gcagcggcctggtgccgcgcggcagccatATGACGAAGCCCACCACCGCGCGGGAAGCC
FzmN_rev	cctttcgggctttgttagcagccggatcctcgagTCAGTAGGCGCCGAAGTACTCACGGTG
FzmO_fwd	gcagcggcctggtgccgcgcggcagccatATGGCGCCTGACCTGCCCGCCCCACCT
FzmO_rev	cctttcgggctttgttagcagccggatcctcgagTCATGTCGATTCCTCTTCCGGTTCCGGCGAT
FzmA_fwd	tttaagaaggagatatacatATGTGCGGAATTGCAGGATTCG
FzmA_rev	gatctcagtggtggtggtggtggtgctcgagTCCGGCGCTGTCGACCGT
pET15b_fwd	CTCGAGGATCCGGCTGCTAACAAAGCCCGAAAGG
pET15b_rev	CATATGGCTGCCGCGCGGCACCAGGCCGCTG
AspB_fwd	agcggcctggtgccgcgcggcagccatatgATGAATACGGATGTGCGTATTGAAA
AspB_rev	cctttcgggctttgttagcagccggatcctcgagTCACTTACGGCCTGCAATG
Kin1K-F	ctggtgccgcgcggcagccatGTGCAGTCGGTCCTCACCGC
Kin1K-R	tcgagtgcggccgcaagcttTTACAGGATCCTTTCGGTTA
Kin1L-F	ctggtgccgcgcggcagccatATGTACTCCCGGTCGTGGT
Kin1L-R	ctcgagtgcggccgcaagcttTCAGAAGGCCTCGAAGTATT
KinM-F	ctggtgccgcgcggcagccatATGATTCCCCGCTATACCC
KinM-R	cgagtgcggccgcaagcttTCAGTCGTCCAGGTCACGAAG
KinN-F	ctggtgccgcgcggcagccatGTGACCTGGACGACTGAGC
Kin1N-R	cgagtgcggccgcaagcttCTACGGGTTGAGCACCCAGGC
Kin1J-F	ctggtgccgcgcggcagccatATGACAAGTTCCGCCGAGC
Kin1J-R	ctcgagtgcggccgcaagcttTCACGAGACCTGCTGCGGC
pET28a-F	AAGCTTGCGGCCGCACTCGAG
pET28a-R	ATGGCTGCCGCGCGGCACCAG
aspB	atgaatacggatgtgcgtattgaaaaagattttctgggcgaaaaagaaattccgaaagatgcatattatggcgtgcaaacgat<br>tcgtgcaacggaaaattttccgattacgggctatcgtattcatccggaactgattaaaagcctgggcattgtgaaaaaaagcgc<br>agcactggcaaatatggaagtgggcctgctggataaagaagtgggccaatatattgtgaaagcagcagatgaagtgattga<br>aggcaaatggaatgatcaatttattgtggatccgattcaaggcggcgcaggcacgagcattaatatgaatgcaaatgaagtg<br>attgcaaatcgtgcactggaactgatgggcgaagaaaaaggcaattatagcaaaattagcccgaatagccatgtgaatatg<br>agccaaagcacgaatgatgcatttccgacggcaacgcatattgcagtgctgagcctgctgaatcaactgattgaaacgacg<br>aaatatatgcaacaagaatttatgaaaaaagcagatgaatttgcaggcgtgattaaaatgggccgtacgcatctgcaagatg<br>cagtgccgattctgctgggccaagaatttgaagcatatgcacgtgtgattgcacgtgatattgaacgtattgcaaatacgcgta<br>ataatctgtatgatattaatatgggcgcaacggcagtgggcacgggcctgaatgcagatccggaatatattagcattgtgacg<br>gaacatctggcaaaatttagcggccatccgctgcgtagcgcacaacatctggtggatgcaacgcaaaatacggattgctata<br>cggaagtgagcagcgcactgaaagtgtgcatgattaatatgagcaaaattgcaaatgatctgcgtctgatggcaagcggcc<br>cgcgtgcaggcctgagcgaaattgtgctgccggcacgtcaaccgggcagcagcattatgccgggcaaagtgaatccggtg<br>atgccggaagtgatgaatcaagtggcatttcaagtgtttggcaatgatctgacgattacgagcgcaagcgaagcaggccaat<br>ttgaactgaatgtgatggaaccggtgctgttttttaatctgattcaaagcattagcattatgacgaatgtgtttaaaagctttacgga<br>aaattgcctgaaaggcattaaagcaaatgaagaacgtatgaaagaatatgtggaaaaaagcattggcattattacggcaatt<br>aatccgcatgtgggctatgaaacggcagcaaaactggcacgtgaagcatatctgacgggcgaaagcattcgtgaactgtgc<br>attaaatatggcgtgctgacggaagaacaactgaatgaaattctgaatccgtatgaaatgacgcatccgggcattgcaggcc<br>gtaagtga

Supplementary Table 3 Buffer conditions used for refolding FzmO. The 216 solutions were made by combinatorially combining each element in a column with one other element from each of the other columns. Conditions were adapted from another study[10].

Buffer (50 mM)	Glycerol	NDSB 256	Salt	Arginine	Glucose
HEPES, pH=7.0	20%	100 mM	300 mM NaCl	800 mM	250 mM
HEPES, pH=8.0	0%	0 mM	100 mM NaCl	400 mM	0 mM
CHES, pH=9.0			100 mM KCl	0 mM	

Supplementary Methods

Synthesis of 5a

Boc-L-glutamic acid 1-*tert* butyl ester (606 mg, 2 mmol, 1.0 equiv.), (benzotriazol-1-xyloxy)tripyrrolidinophosphonium hexafluorophosphate (PyBOP, 1.04 g, 2 mmol, 1.0 equiv.), and *N*-methylmorpholine (220 µL, 2 mmol, 1 equiv.) were dissolved in 8 mL of dry CH_2Cl_2. Acetylhydrazine (163 mg, 2.2 mmol, 1.1 equiv.) was then added, and the reaction mixture was stirred at ambient temperature for 16 h (**Supplementary Figure 9**). The solution was diluted by the addition of 50 mL CH_2Cl_2 and washed successively with 15 mL of 5% $NaHCO_3$, 15 mL of 10% citric acid, water, and brine before being dried over Na_2SO_4. The product was then purified by flash chromatography (silica gel, 15:1 CH_2Cl_2 : MeOH).

^{1}H NMR (600 MHz, D_2O): δ (ppm) = 4.01 (m, 1H, CH), 2.02 (m, 2H, CH_2), 1.92 (s, 3H, CH_3), 1.82 (m, 2H, CH_2), 1.34 (s, 9H, CH_3), 1.31 (s, 9H, CH_3). (**Supplementary Figure 10**). ^{13}C NMR (150 MHz, D_2O): δ (ppm) = 174.11, 173.12, 173.01, 157.59, 83.76, 81.39, 54.00, 29.57, 27.52, 27.05, 26.03, 19.61. (**Supplementary Figure 11**). HRMS: Calc'd for $C_{16}H_{29}N_3O_6$ $[M+H]^+$ = 360.2129, found = 360.2146

Synthesis of glutamylacetylhydrazine (5)

Compound **5a** (48 mg, 0.134 mmol) was dissolved in 2.25 mL of CH_2Cl_2 with 2.5 mL of trifluoroacetic acid, 0.125 mL of water, and 0.125 mL of triisopropylsilane cooled in an ice bath (**Supplementary Figure 9**). The reaction mixture was stirred for 4 h. The mixture was concentrated under reduced pressure and then diluted with 20 mL of water before being washed three times with 8 mL of ethyl acetate. The solution was then dried by lyophilization.

^{1}H NMR (600 MHz, D_2O): δ (ppm) = 3.98 (t, J= 6.3 Hz, 1H, CH), 2.45 (m, 2H, CH_2), 2.13 (m, 2H, CH_2), 1.92 (s, 3H, CH_3) (**Supplementary Figure 12**). ^{13}C NMR (150 MHz, D_2O): δ (ppm) = 173.33, 173.17, 171.14, 52.17, 28.80, 25.08, 19.60 (**Supplementary Figure 13**). HRMS: Calc'd for $C_7H_{13}N_3O_4$ $[M+H]^+$ = 204.0979, found = 204.0988

Synthesis of 6a

Boc-L-glutamic acid 1-*tert* butyl ester (0.909. g, 3 mmol, 1.0 equiv.) was dissolved in 7 mL of dry CH_2Cl_2 with PyBOP (1.56 g, 3 mmol, 1 equiv.) and *N*-methylmorpholine (330 µL, 3 mmol, 1 equiv.) before the addition of hydrazine (141 µL, 4.5 mmol, 1.5 equiv.), and the reaction mixture was stirred at ambient temperature for 15 h **(Supplementary Figure 14**). The mixture was diluted by the addition of 30 mL of CH_2Cl_2 and washed sequentially with 12 mL of 5% $NaHCO_3$, 12 mL of water, and 10 mL of brine before being dried over Na_2SO_4. The product was then purified with flash chromatography (silica gel, 30:1 CH_2Cl_2 : MeOH).

^{1}H NMR (600 MHz, $CDCl_3$): δ (ppm) = 7.82 (br, 1H, NH), 5.38 (d, J=4.2 Hz, 1H, NH), 4.11 (m, 1H, CH), 3.93 (br, 2H, NH_2), 2.22 (t, J=7.3 Hz, 2H, CH_2), 2.12 (m, 1H, CH_2), 1.85 (m, 1H, CH_2), 1.42 (s, 9H, CH_3), 1.41 (s, 9H, CH_3) (**Supplementary Figure 15**). ^{13}C NMR (150 MHz, $CDCl_3$): δ (ppm) = 172.91, 171.29, 155.95, 82.32, 80.00, 53.40, 30.65, 29.35, 28.26, 27.93 (**Supplementary Figure 16**). HRMS: Calc'd for $C_{14}H_{27}N_3O_5$ $[M+H]^+$ = 318.2023, found = 318.2041

Synthesis of glutamylhydrazine (6)

Compound **6a** (0.67 g, 2.1 mmol) was dissolved in 21 mL of CH_2Cl_2, 21 mL of TFA, 1 mL of water, and 1 mL of triisopropylsilane cooled in an iced bath (**Supplementary Figure 14**). The reaction was then stirred for 4.5 h before being concentrated under reduced pressure. The mixture was diluted with the addition of 30 mL of water and washed three times with 10 mL of ethyl acetate. The solution was then dried under reduced pressure.

^{1}H NMR (600 MHz, D_2O): δ (ppm) = 3.72 (t, J=6.3 Hz, 1H, CH), 2.43 (m, 2H, CH_2), 2.06 (q, J=7.24 Hz, CH_2) (**Supplementary Figure 17**). ^{13}C NMR (150 MHz, D_2O): δ (ppm) = 172.50, 172.24, 52.98, 28.68, 25.12 (**Supplementary Figure 18**). HRMS: Calc'd for $C_5H_{11}N_3O_7$ $[M+H]^+$ = 162.0873, found = 162.0875

Synthesis of $^{15}N_2$-acetylhydrazine

NaOH (187 mg, 4.68 mmol, 5.0 equiv.) was added to a solution of $^{15}N_2$-hydrazine sulfate (124 mg, 0.936 mmol, 1.0 equiv.) in H_2O (10 mL). Di-*tert*-butyl dicarbonate (Chem-Impex International, Inc.) (225 mg, 1.032 mmol, 1.1 equiv.) dissolved in dry THF (3 mL) was then added, and the reaction mixture allowed to stir vigorously for 12 h at room temperature (**Supplementary Figure 19**). After 12 h, the reaction mixture was extracted with EtOAc (2 x 10 mL) and the combined organic layers were dried over Na_2SO_4, filtered, and concentrated to afford $^{15}N_2$-Boc hydrazide (83 mg, 66% yield) as a colorless oil. The crude product was used without further purification.

Acetic anhydride (65 μL, 0.681 mmol, 1.1 equiv.) was added to a solution of crude $^{15}N_2$-Boc hydrazide (83 mg, 0.619 mmol, 1 equiv.) dissolved in anhydrous pyridine (10 mL), and the reaction mixture was stirred for 6 h at room temperature. After 6 h, the pyridine was removed under a stream of N_2 gas (**Supplementary Figure 19**). The residue was then purified by silica gel chromatography (4:1 CH_2Cl_2 : MeOH) to afford $^{15}N_2$-Boc-acetylhydrazide (60 mg, 50% yield) as a colorless oil. ^1H NMR (500 MHz, $CDCl_3$): δ (ppm) = 2.00 (d, J=1.5 Hz, 3H, CH_3), 1.44 (s, 9H, CH_3) (**Supplementary Figure 20**). ^{13}C NMR (125 MHz, $CDCl_3$): δ (ppm) = 169.7 (d, J=11.3 Hz), 155.7 (d, J=23.8 Hz), 81.9, 28.0, 20.6 (d, J=11.3 Hz) (**Supplementary Figure 21**). ^{15}N NMR (41 MHz, $CDCl_3$): δ (ppm) = 123.7, 96.2 (**Supplementary Figure 22**).

TFA (5 mL, 67.3 mmol, 198 equiv.) was added to $^{15}N_2$-Boc-acetylhydrazide (60 mg, 0.340 mmol, 1.0 equiv.) dissolved in anhydrous CH_2Cl_2 (5 mL) (**Supplementary Figure 19**). The reaction mixture was then stirred overnight at room temperature. The reaction was concentrated and the TFA removed *in vacuo.* The resulting residue was dried under vacuum to afford $^{15}N_2$-acetylhydrazine (57 mg, 0.300 mmol, 88% yield) as an oily solid. ^1H NMR (500 MHz, DMSO): δ (ppm) = 9.94 – 7.61 (br s, 3H), 1.92 (s, 3H. CH_3) (**Supplementary Figure 23**). ^{13}C NMR (125 MHz, DMSO): δ (ppm) = 169.2 (d, J=10 Hz), 20.5 (d, J=11.3 Hz) (**Supplementary Figure 24**). ^{15}N NMR (41 MHz, DMSO): δ (ppm) = 119.1, 44.5.

Synthesis of $^{15}N_2$-6

To a solution of $^{15}N_2$-Boc-hydrazide (87 mg, 0.649 mmol, 1.0 equiv.) in 10 mL of dry DCM was added Boc-L-glutamic acid 1-*tert* butyl ester (Ark Pharm, Inc.) (196 mg, 0.649 mmol, 1.0 equiv.), *N*,*N*-diisopropylethylamine (284 µL, 1.65 mmol, 2.5 equiv), and hydroxybenzotriazole (105 mg, 0.779 mmol, 1.2 equiv.). The reaction mixture was cooled on ice for 5 min and then 1-ethyl-3-(3-dimethylaminopropyl)carbodiimide (149 mg dissolved in 5 mL of DCM, 0.779 mmol, 1.2 equiv.) was added dropwise (**Supplementary Figure 25**). The reaction mixture was then allowed to stir to room temperature overnight. After stirring overnight, the reaction mixture was concentrated *in vacuo*. The resulting residue was dissolved in EtOAc (20 mL) and then washed with 1 M HCl (10 mL), sat. aq. $NaHCO_3$ (10 mL), and brine (10 mL). The organic layer was dried over Na_2SO_4, filtered, and the filtrate was concentrated *in vacuo*. The resulting crude oil was then purified via silica gel chromatography (1:1 hexanes : EtOAc) to afford $^{15}N_2$-Boc-hydrazide **6a** (201 mg, 74% yield) as a colorless oil.

TFA (5 mL, 67.3 mmol, 140 equiv.) was added to a solution of hydrazide **6a** (201 mg, 0.480 mmol, 1.0 equiv.) in anhydrous DCM (5 mL), and the reaction mixture was allowed to stir overnight at room temperature (**Supplementary Figure 25**). Once the reaction was deemed complete by TLC, the reaction was concentrated *in vacuo*. The resulting residue was then triturated with ether, filtered, and dried under vacuum to afford $^{15}N_2$-**6** (116 mg, 61% yield) as a white solid. ^1H NMR (400 MHz, DMSO): δ (ppm) = 8.41 (br s, 2H), 3.95 (t, J=6.40 Hz, 1H, CH) 1, 2.48 – 2.24, (m, 2H, CH_2), 2.08 – 1.92 (m, 2H, CH_2) (**Supplementary Figure 26**). ^{13}C (125 MHz, DMSO): δ (ppm) = 170.9, 170.7 (d, J=11.3 Hz), 51.7, 28.8 (d, J=11.3 Hz), 25.8 (**Supplementary Figure 27**). ^{15}N NMR (41 MHz, DMSO): δ (ppm) = 118.9, 51.7. HRMS: Calc'd for $C_5H_{11}N^{15}N_2O_3$ $[M+H]^+$ = 164.0814, found = 164.0835.

Supplementary References

1. Du, Y.-L., He, H.-Y., Higgins, M.A. & Ryan, K.S. A heme-dependent enzyme forms the nitrogen–nitrogen bond in piperazate. *Nat. Chem. Biol.* **13**, 836 (2017).
2. Neumann, C.S. et al. Biosynthesis of piperazic acid via N5-hydroxy-ornithine in *Kutzneria* spp. 744. *ChemBioChem* **13**, 972-976 (2012).
3. Matsuda, K. et al. Discovery of unprecedented hydrazine-forming machinery in bacteria. *J. Am. Chem. Soc.* **140**, 9083-9086 (2018).
4. Sugai, Y., Katsuyama, Y. & Ohnishi, Y. A nitrous acid biosynthetic pathway for diazo group formation in bacteria. *Nat. Chem. Biol.* **12**, 73 (2015).
5. Waldman, A.J. & Balskus, E.P. Discovery of a diazo-forming enzyme in cremeomycin biosynthesis. *J. Org. Chem.* **83**, 7539-7546 (2018).
6. Winter, J.M., Jansma, A.L., Handel, T.M. & Moore, B.S. Formation of the pyridazine natural product azamerone by biosynthetic rearrangement of an aryl diazoketone. *Angew. Chem. Int. Ed.* **48**, 767-770 (2008).
7. Gould, S.J., Melville, C.R., Cone, M.C., Chen, J. & Carney, J.R. Kinamycin biosynthesis. synthesis, isolation, and incorporation of stealthin C, an aminobenzo[b]fluorene. *J. Org. Chem.* **62**, 320-324 (1997).
8. Kudo, K., Ozaki, T., Shin-ya, K., Nishiyama, M. & Kuzuyama, T. Biosynthetic origin of the hydroxamic acid moiety of trichostatin A: identification of unprecedented enzymatic machinery involved in hydroxylamine transfer. *J,. Am. Chem. Soc.* **139**, 6799-6802 (2017).
9. Wang, P., Hong, G.J., Wilson, M.R. & Balskus, E.P. Production of stealthin C involves an S–N-type Smiles rearrangement. *J. Am. Chem. Soc.* **139**, 2864-2867 (2017).
10. Vincentelli, R. et al. High-throughput automated refolding screening of inclusion bodies. *Protein Science* **13**, 2782-2792 (2004).
